# Narratives of experiences of violence of Venezuelan migrant women sheltered at the northwestern Brazilian border

**DOI:** 10.1371/journal.pone.0260300

**Published:** 2021-11-19

**Authors:** Maria Y. Makuch, Maria J. D. Osis, Alejandra Becerra, Cinthia Brasil, Helder S. F. de Amorim, Luis Bahamondes

**Affiliations:** 1 Faculty of Medical Sciences, Department of Obstetrics and Gynecology, University of Campinas (UNICAMP), Campinas, SP, Brazil; 2 Center for Research in Reproductive Health of Campinas (Cemicamp), Campinas, SP, Brazil; 3 Faculty of Medicine of Jundiaí, Department of Collective Health, Jundiai, SP, Brazil; 4 Direction of Basic Attention Care, Health Secretary, Municipality of Boa Vista, Boa Vista, RR, Brazil; 5 Direction of Basic Attention Care, Health Secretary, State of Roraima, Boa Vista, RR, Brazil; Murcia University, Spain, SPAIN

## Abstract

**Objectives:**

To know the experiences of Venezuelan migrant women living in shelters in Roraima state at the northwestern border between Venezuela and Brazil regarding situations of violence as part of the dynamics of everyday life.

**Materials and methods:**

Data were collected in January 2020 through 12 focus group discussions (FGDs) with 5 to 14 Venezuelan migrant women aged 18–49 years old living transitorily in five shelters established by the United Nations High Commissioner for Refugees (UNHCR) and the Brazilian government. We obtained individual and shared views on the experiences regarding violence that migrant women may experience in their everyday life. To organize the FGDs, variations in age and the time women were living at the shelters were considered. All FGDs were held in a place at the shelter that guaranteed privacy and secrecy so that women could express themselves freely. The initial question was broad and open ended and was followed by more specific questions about situations of domestic violence and other types of violence.

**Results:**

The main themes identified were the following: i) women’s perceptions on domestic violence, ii) women’s perceptions on how humanitarian organizations were managing the episodes of domestic violence, and iii) situations considered violence in everyday life at the shelters. The FGDs showed that the reported violence inside the shelters was high, and several forms of violence emerged. Violence was identified as physical aggression and psychological threats, and violence in everyday life at the shelter included xenophobia when the migrants went outside the shelters that was perceived and described as violence.

**Conclusions:**

According to the perspective of Venezuelan migrant women violence was part of everyday life among those living in the UNHCR shelters at the northwestern border of Brazil-Venezuela. These women are not comfortable with this situation, and it is difficult for them to understand and handle the episodes of violence.

## Introduction

It is estimated that 272 million people worldwide have been forced to leave their homes in search of new opportunities and to meet their basic needs [1,2]. Migration has an important effect on the lives and health of people and can increase inequalities and the exposure mainly of women and adolescents to situations of violence [3]. In 1993, the UN general assembly for the Human Rights Committee on the Elimination of Discrimination against Women (CEDAW) defined violence as follows: “*any act of violence that has or may result in physical*, *sexual or psychological harm or suffering for women*, *as well as threats of such acts*, *coercion or arbitrary deprivation of liberty*, *whether they occur in public life or in private life*” [4].

A high prevalence of violence was reported among migrant women [5]. It is estimated that 48% of migrants are women, and it has been reported that violence inflicts serious damage to physical and mental integrity [6]. Furthermore, it was reported that 1 out of 3 women worldwide (35.6%) is a victim of violence [7,8]; additionally, in American countries, 29.8% of cases of violence are committed by an intimate partner, and 10.7% are committed by a non-intimate partner [6,9].

During the migration process, different forms of violence and gender inequity mark the trajectory of many women. The occurrence of social violence, poor quality of life due to economic limitations, lack of employment, homicide, and sexual assault, among others, are the difficult issues women have to live with, and these issues increase their vulnerability at the time of migration [10]. In this context, women may be victims of rape by assailants or forced sex by traffickers [11]. Additionally, xenophobic populism makes the integration of migrants into a new society difficult and can be transformed into a barrier to healthcare [12] by increasing the exposure to risk factors such as insecurity (undocumented registration), language barriers, economic difficulties, labor abuse [13–15] and limited access to education and public health care [11,16].

Among migrant women, the main perpetrators of violence are people who are close to the assaulted women, and the situation of displacement could aggravate previous abuse. In shelters, the perpetrators could be intimate partners, relatives or acquaintances or military and police forces, and perpetrators outside the shelters could be strangers who take advantage of their vulnerability [17,18]. Violence encompasses the absence of a woman’s right to refuse forced sex, and it was reported that many migrant women have suffered some kind of violence by their partner [19–25].

After episodes of violence, there could be short- and long-term consequences that affect sexual and reproductive health (SRH) such as unplanned pregnancy, sexually transmitted infections (STIs), human immunodeficiency virus infection (HIV), depression, anxiety, and post-traumatic stress disorder [17,20,26].

One of the responses to the migration crisis is the Minimum Initial Service Package (MISP) developed by the Interagency Working Group in Reproductive Health (IAWG) [27] to identify, prevent and treat the consequences of violence among other SRH issues. Even though this is a great step in the direction of improving healthcare for migrant women, the suffering of victims of violence is increased by the limited availability of multidisciplinary SRH services that offer medical and psychological care for victims of violence and women’s lack of knowledge regarding SRHR [14,28,29].

As of 2019, five million Venezuelans have been displaced due to economic and social crises, mostly migrating to neighboring countries: Colombia, Peru, Ecuador, Argentina, Chile, and Brazil. There are still few reports regarding the impact that this migratory process has imposed on women, men, children, and families. A recent report [30] regarding violence among Venezuelan migrants living in Colombia offers information to initiate this discussion.

The state of Roraima on the northwestern Brazil-Venezuela border has become the main Brazilian gateway for Venezuelan migrants. Roraima is one of the smallest and the least populous Brazilian states, having a low population density (615,000 inhabitants), and is among the states with the highest poverty rates at the national level [31]. Since 2016, an average of 600 Venezuelan migrants have entered Brazil through this border daily, posing challenges for the region to manage this constant incoming number of people. The Brazilian government has been transferring migrants to Boa Vista, the state capital, as initial settlements [1,32,33]. As a response to the crisis originated by this migration, the United Nations High Commissioner for Refugees (UNHCR), in cooperation with the International Organization for Migrations (IOM), the Brazilian federal government and the Brazilian army, established 13 shelters in Roraima in the context of a “welcoming operation” (*Operação Acolhida*) as a transit settlement before they will be reallocated to other Brazilian cities.

Due to the need to improve humanitarian assistance and the still scarce information available on violence among Venezuelan migrant women, our aim was to know the perception of Venezuelan migrant women living in shelters at Roraima state at the northwestern border of Brazil-Venezuela, regarding situations of violence as part of the dynamics of their everyday life.

## Materials and methods

### Study design and participants

We are reporting data on violence from a study on SRH issues conducted with Venezuelan migrant women transitorily sheltered in the city of Boa Vista, the capital of Roraima state, Brazil, who were waiting for reallocation to other parts of the country [33,34]. In this paper we present data from the qualitative arm of the study on domestic and everyday violence. The study design and instruments were adapted from the MISP readiness assessment tools from the IAWG on Reproductive Health [27]. The research protocol was approved by the Ethics Committee of the University of Campinas, Campinas, SP, Brazil, and all participants signed a written informed consent prior to participation in the study.

A qualitative phenomenological descriptive study [35] was carried out in five shelters in January 2020. We collected data through 12 focus group discussions (FGDs) to obtain individual and shared views on experiences of violence among migrant women in their everyday life at the shelters [36]. The participants of the study were Venezuelan migrant women aged 18–49 years old, and variations in age and the time the women were living at the shelters were also considered. These criteria were adopted to obtain a diversity of experiences and views of the participants. We excluded: i) women aged over 49 years old because we only considered women at reproductive age and ii). adolescents under 18 years old were excluded because according to the Brazilian law they only can participate in research studies after their parents or legal guardian signs an inform consent and because we consider that adolescents under 18 years old may present different life experiences. Before the beginning of the study, it was defined that at least two FGDs would be carried out in each of the chosen shelters, and the final number was confirmed during fieldwork through data saturation. There was consensus among the 3 senior researchers (MYM, MJDO, and LB) that the data were significant for the objectives proposed for the study and that no new information was emerging from the FGDs. The Standards for Reporting Qualitative Research were followed [37].

### Data collection

The FGDs were organized in a manner such that they did not interfere with the regular activities of the shelters and were at convenient times for the women. General information on the research and on how FGDs were being organized was provided by the staff of the humanitarian agencies to the women in each shelter, and the researchers explained the objectives of the study and how FGDs would be conducted to those women who were interested in participating. All FGDs were held in a place at the shelter that guaranteed privacy and secrecy so that women could express themselves freely. No members of the staff of the humanitarian organizations participated in the FGDs, nor did they have access to the place where the FGDs were held. The participants did not receive financial compensation. The FGDs had between 5 and 14 women participating.

The FGDs were conducted using a semi structured guide (Supplementary material). The initial broad and open-ended questions “*Please tell me about your experience of living in a shelter*”; were followed by more specific questions regarding SRH issues including experiences of domestic and other types of violence in everyday life after migration, at the shelters and in the surrounding community outside of the shelters. The present report referred to the findings on women perceptions on violence. Open-ended questions and polls were used to ensure feedback from participants and facilitate further discussion. All the FGDs were conducted by two social scientists, a psychologist and a sociologist (MYM and MJDO) with large experience in qualitative studies and in SRH. The FGDs were recorded digitally and ranged between 60 to 100 minutes. All audiotape data and transcripts were saved in a password-protected computer. Prior to the initiation of each FGD, the participants filled out a characterization form with some sociodemographic characteristics.

### Data analysis

Conventional thematic analysis of the manifest content using an inductive approach was carried out [38]. The audio recordings of the FGDs were transcribed by an independent qualitative transcription specialist. Then, the transcripts were checked by two researchers (MYM and MJDO) against the recordings for accuracy and integrity. In the first stage of the analysis, the interviews were read, and the salient topics, ideas, experiences, and recurring behavioral patterns were highlighted in the transcripts. Subsequently, the interviews were organized to identify the significant themes that emerged from the data. These themes were identified and coded manually in the transcripts independently by two of the authors (MYM and MJDO) and reviewed by another author (LB). The codifications were compared, and the discrepancies were discussed by all the authors. All data reported in the results section were included upon consensus of all the authors. This process was followed to ensure a conscious and transparent process of describing the emerging data.

**Fig 1:**
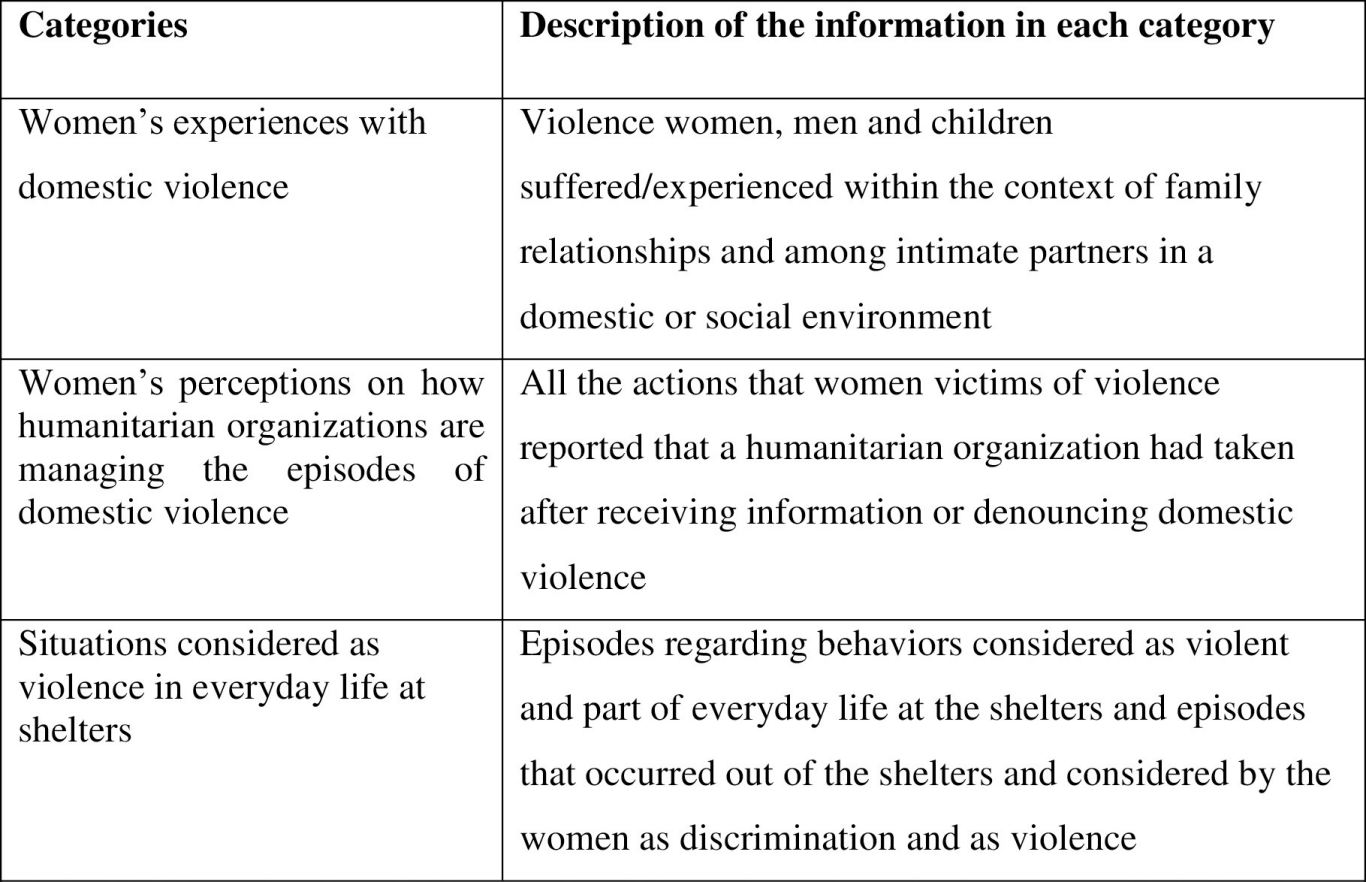
Definition of the categories of analysis.

Qualitative data analysis software NVivo (version 12, 2020, QSR International Pty Ltd.) was used to implement the data management, coding process and analysis. The FGDs were conducted in Spanish, and the quotes were translated by one bilingual author (MYM) into English and back-translated into Spanish by another bilingual author (LB) to ensure that the original meaning was not changed. The main themes identified were organized into three categories of analysis: i) women’s perceptions on domestic violence, ii) women’s perceptions on how humanitarian organizations are managing the episodes of domestic violence and iii) situations considered violence in everyday life at shelters (Fig 1).

## Results

A total of 111 women participated in 12 FGDs. Table 1 shows some sociodemographic data of the participants. The mean (± SD) age was 34.4 (± 11.8) [range 18 to 49] years old, and the time they reported they were living at the shelters was 5.5 (± 4.9) [range >1 to 30] months. After the first broad open-ended question the participants in all FGDs engaged in the discussion and narratives of diverse situations of violence including physical aggression and psychological threats and violence in everyday life at the shelter.

**Table 1:** Some sociodemographic characteristics of women who participated in the focus groups discussions (n = 111).

Age (Mean ± SD) (years)	34.4 (± 11.8)
Children with the migrant woman (Mean± SD)	2.5 (± 1.1)
***Ethinicity (n = 108)***	N (%)
White	25 (23.1)
Black	16 (14.8)
Biracial	60 (55.6)
Asian	7 (6.5)
***Educational level* (n = 110)**	
Illiterate	2 (1.8)
Primary	16 (14.5)
High school	61 (55.4)
Postsecondary school	31 (28.3)
***Migrated with (n = 109)***	
Children and a partner	39 (35.7)
Only with children	35 (32.1)
Only with a partner	4 (3.6)
Children and other relatives	13 (11.9)
Alone	9 (8.2)
Only with other relatives	9 (8.2)
***Cohabitation status (n = 104)***	
With a partner	63 (60.6)
Without a partner	41 (39.4)
***Paid work (n = 111)***	
In Venezuela	99 (89.1)
In Brazil	8 (7,2)

### Women’s perceptions on domestic violence

In all FGDs, the women discussed domestic violence as a common situation at the shelters. They referred to frequent episodes of physical aggression, psychological threats and verbal aggression practiced by men, both husbands and intimate partners, against women. They also discussed the strategies that women used to handle domestic violence. Among these strategies frequently observed by women were the minimization of the violent episodes, not to admit that the violent situation had occurred, and the adoption of attitudes and actions to maintain the violence in their private sphere. They also discussed the opposite situation in which some women denounced domestic violence to the authorities of the shelter.

*“There are women here (at the shelters) that are abused …*. *And they don’t denounce*, *they continue with their partner*, *they continue with their aggressor*. *I am in a similar situation*. *I was abused by my husband*, *but then I migrated*, *and I feel safe here …*.*”* FGD #9

*“Yes*, *there is also verbal (violence) … in the tent blah blah blah the discussion*, *the tent shakes but nothing is known out of the tent*. *We ask the women*, *did he hit you*? *Was he violent*? *And they don’t respond …it’s a lie*.*”* FGD #12

In the narratives women referred to situations in which men and women engaged in relationships other than with their stable partner. In their perceptions a frequent reason for the occurrence of these relationships within the setting of the shelters were that people lived close one to another, that there was a lack of privacy, and that this facilitated contact among men and women. However, many discussions regarding these relationships were in the context of stereotypical situations and behaviors. In that direction were the discussions that some women did not wear appropriate clothes and that they used provocative clothes to attract men’s attention and try to establish relationships with them; therefore, they were in some way responsible for the behavior of men. Additionally, there were similar discussions that some women felt jealous and had attitudes that provoked violent reactions from their partners. They discussed this kind of behavior in what they called the “*Latin American machismo*”, and in their opinion, many men considered it their right to be in a relationship with women other than their stable partner.

Furthermore, there was much discussion on the situations when women did not comply with the domestic roles expected of them and were in some manner responsible for the violent reactions of men. Furthermore, it was also frequently discussed that many women considered it their obligation to take care of the children, household chores and satisfy all the needs and desires of their stable partner, independent of their attitudes and behavior.

*“Since in Latin American communities’ men are very ‘machista”*, *women have to take care of the children and of the house chores; and if they don’t have children*, *women are in a bad situation; men don’t treat them well*, *and if you don’t obey …*. *Then*, *there are the situations where they (men) have another woman out of the house … and when they bring the other one here (to the shelter)*, *that’s when the worse situation starts*. *It is like a world war because it’s the woman against the man*, *the wife against the other woman*, *the other woman against the wife…*.*”* FGD #4

There was also some discussion of episodes in which some women were violent (physically, emotionally, and verbally) with their partners. Some of these women threatened their partners that they would denounce them as the aggressor as a strategy to be in control of the situation. This behavior intimidated some men who were unable to react because they were afraid that the authorities of the shelter would consider the men the aggressors. Some women used the rule that men who are violent with their partners must abandon the shelter to threaten their partners.

*“The problem is the following*: *Venezuelan women are feisty*, *and Venezuelan women have a motto*: *if you are going to hit me*, *try to do it in such a way that I will not get up*, *because if I stand up again I will crack your head*, *and afterwards*, *I will go to the police station and say that I killed you in self-defense*. *That is quite common in the Venezuelan culture…*.*”* FGD #4

Some participants discussed that some women at the shelters who had knowledge that the Brazilian law protected women who were involved in violent relationships with their partner. This knowledge empowered them, and they used the information as a strategy to control their violent partner and to protect themselves.

*“I have been here for a year and a half*, *and we never had that problem that he would hit me … no*, *never*. *I always tell him*: *remember*, *you are not in Venezuela*, *and here in Brazil*, *women are heard and are protected*. *Here*, *women have a law that is called ‘Maria da Penha’*. *I bet that if I scream here*, *the military personnel will come and beat you*. *“*FGD #6

Within the context of domestic violence, women frequently referred to situations of men being physically and verbally violent with their children, including small children. When women referred to parental violence, some of them were quite uncomfortable and even angry with this situation. Particularly, participants showed unease with situations where they considered that mothers and fathers should protect their children instead of adopting violent behaviors with them. Some stated that they had denounced these incidents to the authorities of the shelter.

*“There is also much violence against the children; there are many mothers that hit the children with what they have on hand*, *a cable*, *or with whatever they have; they take off their cloths and beat them*. *There are many people here that are problematic … I have tried to interfere a couple of times in these situations*, *and my husband always says*, *don’t interfere…*.*”* FGD #9

### Women’s perceptions on how humanitarian organizations are managing the episodes of domestic violence

The participants reported that when episodes of domestic violence practiced by men came to the knowledge of the organizations that administered the shelters, the aggressors were asked to abandon the shelter and lost the benefits of humanitarian assistance. Another strategy that the humanitarian organizations used to manage situations of violence was to transfer the aggressors to another shelter, depending on the type and severity of the incident.

*“There was a case here that a young man was asked to leave the shelter*. *He was aggressive and beat his partner and also his mother*. *And the case was very difficult*, *and he was removed from the shelter*. *Afterwards*, *his wife also left*, *but his mother is still here*.*”* FGD #11

Women also discussed that in the past, when the shelters were being organized and during the initial phase of their functioning, the management of domestic violence was different. Male aggressors were referred to psychological assistance and had the possibility to continue in the shelter with their partners and families. However, that possibility is not available anymore. Participants considered it important to provide this kind of assistance again.

*“Some time ago there was another option*. *A man that was violent with his partner had to go to five sessions with a psychologist*. *After he finished his sessions*, *he could go back to his family and live with them again and stay at the shelter with his wife and family*. *But recently*, *in December 2019*, *in the days of community discussion*, *we heard them (the humanitarian staff) say that those who have to leave the shelter because of domestic violence can’t come back…*.*” FGD* #6

Situations in which women protected a violent partner were also mentioned, and the discussions evidenced different attitudes regarding this situation. Some women were ambivalent to denounce their violent partners because they felt guilty, others repented after they denounced their partners, and some interceded in favor of their partner because even though their partner had been violent with them, they did not want their partner to suffer the difficulties of living outside the shelter.

*“There are some women that denounce their husbands for being violent*, *and after a couple of days they say*, *‘my husband wants to come back and live with me*,*’ and they withdraw the charges*.*”* FGD #9

In one of the FGDs, the women discussed a situation in which some male aggressors manipulated the facts to present the women as responsible for the violence and the aggression. In those cases, according to the participants, the humanitarian organization did not take the side of the woman who was assaulted by their partner and did not protect them. Some women expressed that in their opinion, there was not enough time and effort dedicated to the many and diverse issues of violence that occurred in the shelters.

*“Here*, *there is no control*. *There was a family here where the man started to have a relation with another woman*. *And the wife who was hurt and frustrated asked him to resolve this situation*. *So the two women started to fight*, *and the man*, *instead of separating them and taking the side of the wife*, *held her for the other woman to beat her*. *She was red like a tomato … here*, *the organization (of the shelter) did not give her the protection she needed*. *They left her in this shelter and sent him to another shelter where the other woman was*. . . .*”* FGD #10

### Situations considered violence in everyday life at shelters

Participants also referred to other situations in their everyday life that in their perceptions were a form of violence and occurred due to living conditions in the shelter. Many everyday routines or activities that women considered were in their private sphere were frequently exposed publicly because of scarce privacy and exposure of intimacy. Further, they reported that they did not know of any actions to improve these life conditions. According to their perceptions, some situations could be improved if women were more actively included in the process of decision making regarding some routines and improvements in the shelters.

*“I am constrained and frequently scared of using the toilets because there are some men always around that area*, *and their faces are scary … my God*, *you don´t feel comfortable going to the toilet…*.*”* FGD #2

Robberies were reported to be constant events in the shelters and a situation that caused much unease. This situation was considered a form of violence practiced against people who were already living in vulnerable conditions. Women reported that personal belongings were robbed from the tents when residents were distracted or engaged in other activities. Another strategy described for these robberies was the initiation of an aggressive situation as a distraction. According to the participants, these situations were frequent and therefore required constant attention to what was happening in the tents and with their personal belongings.

*“I arrived here in Brazil about 15 days ago*, *and already they robbed me*. *I felt terrible because I was assaulted physically by Venezuelans because they were not Brazilians*. *And I thought*, *oh my God I can’t believe that in Brazil I was robbed by Venezuelans… unfortunately*, *my neighbors stole my clothes*, *my children’s toys; they stole almost all the things I had*. *That is why I asked to change to another place in the shelter*, *and the neighbors that I have now are calmer*, *and they don’t steal*.*”* FGD #10

The behaviors that some migrants adopted in the refectory were described as violence. Rules of the refectory established that residents had to be in line and that women, children, elderly persons and persons with special needs were the first to enter, receive food and have access to the tables. However, there were some men who did not wait in line and pushed their way to first place.

*”… because some men want to go in first before women and children*, *and that is not correct*, *that is not what has been established*. *Well*, *some men don´t want to wait*, *don´t want to wait for their turn to go in*.*”* FGD #5

Also, women considered violence the attitudes of some residents who had been living for a longer time at the shelters when they imposed unwritten rules on the newly arrived residents as a way to make it clear “*who had the leading voice*”.

*“In the shelter*, *there is a big problem with those who arrived first*. *They believe they are the owner of the place*, *and then those who just arrived receive worse things and are not well treated*.*”* FGD #2

Women also reported that fights and aggression among the migrants living at the shelters were more frequent on weekends due to alcohol abuse, prohibited in the shelter; however, there were people who found ways to bring it in.

*“Because they drink*, *some are conscious*, *and others are not … but I hear comments that there is drinking here*. *I don’t know how they manage to bring it in because there are controls*.*”* FGD #5

The women referred to episodes were Venezuelans living at the shelters were victims of verbal and physical aggression when they were out of the shelters, in the community looking for a job, buying something at a market, or asking for help with their needs. In one of the FGDs, women concluded that these attitudes were because some Brazilians living at the border of Brazil and Venezuela had experienced unpleasant and negative situations; and for that reason, all migrants were considered unreliable persons.

*“There are many occasions in which we have to leave the shelter and do things on the streets; we have to look for something we need or to look for a job*. *And we are at risk because there is much xenophobia against Venezuelans*. *There was a man in a car*, *and he tried to run us over*. *If I wouldn’t have pushed my son*, *he would have run him over*.*”* FGD #9

*“You go to a market to buy something*, *and immediately*, *you have somebody by your side to see if you are taking something*. *You look at the products and you put them back and you have somebody from security by your side … it is a quite delicate situation*, *you can’t go near the market*, *look at the things*, *touch them*. . . .*”* FGD #9

## Discussion

Our findings of this qualitative study broaden the knowledge on the violence that Venezuelan migrant women living in UN shelters in Brazil suffer in their everyday life. We gave voice to women to discuss their perceptions regarding violence that they themselves may have suffered or that they observed that other women living at the shelters were experiencing. At the time of the study, the participating women were living immersed in a situation of uncertainty regarding their future and restricted mobility.

Living at the UN shelters offered migrant women the opportunity to have a transitory place to stay, resolved their basic needs in this vulnerable situation and facilitated the process of documentation and the possibilities of resettlement to other regions of Brazil. However, it also posed a time of discontinuation in their life projects and a new organization in their everyday life [39]. In addition, the migrant women were living in a community of people, some with different life habits and social behaviors; were subject to rules; and had scarce privacy. The administration and discipline organized by community shelters are not always understood and accepted by all migrants, and conflicts may arise between them [39].

Recognition that some kind of attention is important for women victims of domestic and other forms of violence may be a determinant for the future of these women, the couples and the families involved in these violent episodes and may be the first step towards action. Frontline humanitarian workers should be aware of the existence of these violent episodes and of the quantity and intensity with which they occur and should be trained to handle these situations properly [33,40].

Based on the WHO definitions of violence [6,9], it was possible to distinguish two main types of violence present in the everyday life of women in the narratives of the participants of the FGDs: domestic violence and community violence. We identified domestic violence as all violent episodes practiced by intimate partners and violence against their children. The women during the FGDs discussed that those living at the shelters sought to keep all the violent episodes that occurred between the couples or with the children in the private domain. Most likely, the facilitators of the FGDs created an adequate atmosphere within an environment with privacy that encouraged women to talk more freely about episodes of violence.

Domestic violence is a complex phenomenon related to the social and cultural values of the people involved. In this context, especially in societies where the traditional pattern of gender relations prevails, it is common for women who suffer this type of violence to want to keep the situation private as a personal and family matter, as they understand that making this situation public will bring shame for her and for the family. Another fear underlining women’s silence is the fear of being abandoned by her partner who will also be dishonored by making this situation public [41]. Other authors also described this behavior [42], which has been associated with a common belief that if women are punished and experience violence from their partners, it must be because they have done something that is wrong or that makes them unworthy of respect and consideration. A study carried out in Venezuela [43] also discussed the tendency of some women to naturalize male dominance and control because for them, it is a normal behavior and part of the relationship.

Further, it was described [44] that one of the significant predictors of sexual coercion and physical violence are attitudes of men who like to be in control of the relationship and their partner. One of the components of this type of relationship is jealousy, which has been nominated as romantic jealousy which is different than other type of jealousy like for example sibling rivalry. Various emotions such as anger, frustration, insecurity, shame, humiliation among others were described as emotions that there are parts of romantic jealousy. Romantic jealousy based on real or imaginary facts may create conflicts which could derive in IPV [45].

These gender stereotypes present in many settings, especially in some Latin American scenarios, are strongly rooted in the social representations of gender roles. The women discussed in the FGDs the nominated *Latin American machismo*. We used the term *machismo* to refer to a gender role assumed by some Latino men, linked to traditional gender and masculine roles. Within this role it is considered that men should be strong, virile, providers and dominant; and they expect women to be passive. These men believe that they have sexual freedoms including a right to extramarital relations [46].

We cannot affirm that the episodes of violence discussed by the participants during the FGDs were exclusively due to the fact that they were living in a precarious situation. WE have not information regarding the occurrence of violence before they migrated. This is an issue which needs more in depth research. However, what seemed evident was that the episodes of IPV acquired a great relevance in the day-by-day life in the shelters for these women. We can speculate that this was due to the fragility of their situation.

Migrant women feel more isolated in the situation of domestic violence, frequently with language barriers, a lack of knowledge regarding the services that may help them in this situation, and no knowledge of the ways to access these services [29,41]. Additionally, they are uncomfortable with the possibility of misunderstandings with their cultural and religious beliefs [47]. We can presume that in the case of Venezuelan migrant women living in shelters, there may have been an additional reason for not revealing the domestic violence they had suffered. They reported that men who were violent, when denounced, were at risk of having to leave the shelter and lose the social support from the humanitarian organizations and the community of Venezuelans living at the shelter. It could be that women in this particular situation became more prone to feel guilt if they denounced their partners.

It became evident that women had knowledge of the Brazilian law regarding violence that punishes men who adopt these behaviors [48]. This knowledge of the law and the rules of the shelters were used by some women as a strategy to control the violent behavior of their partners. This situation was different than the findings of a study with Venezuelan migrants in Colombia who reported not being fully aware of the local laws regarding sexual violence and IPV [29]. This difference in knowledge may be because the women in our study were living in UN shelters; in addition, in Colombia, most women reported an irregular legal status.

The perception women had on how the humanitarian organizations handled the episodes of violence been diverse. There were satisfactory experiences with the interventions and many comments on the lack or inefficiency of the actions taken [1,40,49]. According to the narratives, it seemed that women liked the former strategy implemented at shelters to assist aggressors through group activities, considered important and useful interventions, to prevent this kind of action. The shift of the former strategy regarding the management of domestic violence from psychological intervention to removing violent men from the shelter to live on their own was a radical decision. We can suppose that this was due to the increased number of migrants in a short time and created difficulties in maintaining this strategy, which may have overwhelmed the humanitarian organizations and the local collaborating institutions impacting the implementation of some interventions. In Brazil, there are few programs aimed at men who perpetrate violence against women, although this is a problem that is reaching alarming proportions [4]. Specifically, in the state of Roraima, there is no information about institutions involved in this matter [50].

Violence against children was considered an unacceptable behavior, and women were shocked when they observed episodes of physical violence of some mothers upon their children. This kind of violence practiced by caregivers or family members is among the most prevalent types of episodes of violence that children suffer during the migratory process. It was reported [51] that children during migration have suffered physical violence; furthermore, in general, the reviewed studies did not present detailed information about the type of violence the children suffered and did not discriminate by gender, the percentage of children that suffered violence, and the characteristic of the aggressors.

In the context of domestic violence, aggression against children in the communities of migrant families has been linked with parents’ emotional problems related to the condition of migrants. A Peru-based study with migrant mothers identified that domestic violence against children was associated with depression, a decrease in interest and compromises in the care of their children, less warmth in the relationship with their children, and less energy to invest in their parental role [52]. Additionally, domestic violence was associated with behaviors that children internalize and develop. Considering this association, it is evident that the impact of domestic violence may impair children’s healthy development and compromise the possibility of better living conditions in the new country. It is important to state that the search for better living conditions for themselves and their children has been one of the main motivations for migration reported by adults. These results provide evidence in favor of adequate interventions for migrant families regarding the relationship between parents and children, the importance of relationships free of violence, and strategies to help parents develop better parenting skills and to help these adults take care of their mental health [51].

In our study, the women referred to violence suffered outside the shelters in the community when they discussed episodes of verbal aggression they had suffered from the local population. Similar results were found in a Colombian-based study with Venezuelan migrants who reported experiences of xenophobia and psychological and physical violence as part of their interaction with the local community [30]. According to studies conducted in other countries, attitudes of xenophobia towards immigrants may be related to fear of the actions of the migrants and of losing jobs and opportunities by the nationals, even if there is no concrete or solid evidence to support those fears [53]. Xenophobia may constitute a barrier for migrants to seek and obtain access to public health services and the health care they need [54] and at the same time is quite complex to handle because it is rooted in social feelings.

Our study has strengths and limitations. The strengths are that we analyzed women’s narratives about their experience [36–38], and they could freely express their perceptions about situations of violence. This methodological approach gathered data that showed how complex and sensitive it is to discuss domestic violence and violence as part of everyday life. In addition, the FGDs allowed us to obtain information about experiences, perceptions, and beliefs from the interaction of the participants [36]. A possible limitation may be that the study was developed in only one setting of Venezuelan migrant women staying for a transitory time in UN shelters after crossing the border.

Our results reveal some evidence on how and why this problem may not become evident if we are not aware of it and do not investigate and respect the evidence and the consequences of violence in the lives of migrants with much care. There was an important tendency among migrants to keep situations of violence within the private sphere of the people involved.

Our study contributes to broaden knowledge providing insights regarding Venezuelan migrant women in Brazil which could help stakeholders and policy makers to take action to reduce violence which is an important issue of SRH. Healthcare providers, stakeholders, and everybody involved in humanitarian activities should be aware of the existence of violence, of the characteristics of the population, and the meanings these situations of violence have for all the involved. This knowledge should be part of the training that professionals and humanitarian agents receive to handle situations of violence experienced by women, children, men, and families within these settings. Moreover these results could be used to organize educational activities and support groups on violence to help women and men identified and deal with situations of violence. More studies and evidence are required to understand not only the existence and magnitude of the problem but also the depth of the effects that domestic violence has on women, children, and families. This understanding should lead to more actions to adapt responses and strategies to handle the violence present in everyday life in humanitarian settings.

### Conclusions

In conclusion, we observed that according to the perspective of Venezuelan migrant women violence was part of everyday life among those living in the UNHCR shelters at the northwestern border of Brazil-Venezuela. Nevertheless, we cannot confirm that many of the reported violent episodes were not occurs before they migrate to Brazil, and violence was more associated to these groups independently of the place of residence. Further, these women are not comfortable with this situation, and it is difficult for them to understand and handle the episodes of violence.

## Supporting information

S1 File Supplementary material FGD English Spanish PLOS ONE. (DOC)
