# Supplementary material for: Narratives of experiences of violence of Venezuelan migrant women sheltered at the northwestern Brazilian border
Source: PLoS One. 2021 Nov 19;16(11):e0260300. doi: 10.1371/journal.pone.0260300 (PMC8604314; doi:10.1371/journal.pone.0260300)
Supplement: S1 File — (DOC) [file pone.0260300.s001.doc]

**ENGLISH**

**PROJECT: "SEXUAL AND REPRODUCTIVE HEALTH SERVICES FOR VENEZUELAN WOMEN ON THE BORDER BETWEEN VENEZUELA AND BRAZIL - 2019/2020".**

**Focus Group Discussions –Guide Instrument**

**Women (18-49 years old)**

**INTRODUCTION**

Before starting the focus group discussion (FGDs), be sure to receive the informed consent form signed by the participant.

"Hello, my name is _______________. I work for CEMICAMP. This is my team ____ [Say the name of the colleague (note taker)]. We are conducting research on issues related to sexual and reproductive health in this humanitarian crisis. The purpose of the FGDs is to learn more about the integration of reproductive health into the humanitarian responses you are receiving, as well as, your experiences of accessing services. During the discussion, I will ask you questions about your experiences with reproductive health care. My colleague will write down some observations and if you agree we will be recording the answers. Your answers will be used for a report that may be published or presented in one or more public health forums. Your name will not be included in any document or presentation, but the name of the place where we are located may appear. There is no direct benefit to participating in this study. If you are uncomfortable with any of the topics addressed in our conversation, you may not participate or stop participating at any time during the discussion. You may also choose not to answer any of the questions.

***Can we continue?***

**QUESTIONS**

- **First, we would like to ask you some general questions about the situation of migrant women here.**

A1. What problems are of most concern to the women who are living here?

A1a. What can be done to improve these particular problems?

A2. Please can you tell us how the organizations which responding to this emergency have communicated with the affected community regarding the emergency response? How was the contact with you? How did you learn about it?

- In your opinion, was the form of communication adequate, sufficient, did you understand it?

- Are the centers where you go for healthcare provided by the organizations providing humanitarian aid/services or by the municipality?

- Now, we would like to ask you some general questions about the assistance of health services here where you live now.

B1. Since you arrived, what supplies for menstruation, hygiene or clean delivery have been distributed to women and girls?

**In case that at least some of these supplies were distributed:**

B1a. Who did the distribution, where was it done and how many times was it done in the last month?

B1b. What do you think about these distributions? What does the community in general think about these distributions?

- Are they sufficient or not?

- Who decides the amount?

**If at least one of these supplies was not distributed, please inquire:**

B1c. What reasons have you been given for not having these supplies?

**Continuing the discussion on the quality of healthcare services in Boa Vista.**

B2. How many women have been involved in the organization and delivery of services to encounter health needs in this shelter?

B2a. With whom can you discuss whether there are unmet needs of girls/women in this shelter (e.g., block leader, shelter leader, women's association, NGO, UN, etc)?

B3. Have you had contact with healthcare services in Boa Vista? How have the health personnel treated you?

B3a. Have you known or heard about the experiences of other women who have had contact with healthcare services in Boa Vista?

B4. What programs are there for adolescents? [Probe: have you ever heard of/visited a program specifically for youth?]

B4a. What reproductive health services does this program offer?

B5. What are some of the reasons a woman would not look for health services?

B5a. (Probe: Any problems with clinic or hospital hours, practitioners or medications, the woman's age or marital status?)

B6. What safety precautions are barriers to women looking for healthcare services?

B6a. What are the things that concern women about the comments they hear?

B7. In general, how do you think healthcare services for migrant women and youth can be improved?

**We would also like to discuss healthcare, especially sexual and reproductive health. Let's start by talking about issues related to pregnancy and childbirth.**

B8. When women are pregnant, where do they seek medical care?

B8a. How did you learn about these services for pregnant women and for giving birth? Do you know how prenatal care works? Do all pregnant women do prenatal care?

B8b. What have you heard about the quality of services for pregnant and birthing women?

B8c. What symptoms would cause women to seek help when they are pregnant? (Probe: health problems, danger signs) How do they get help? (appointment, tests)

B9. Where do women seek medical care when they are about to give birth? How do they get there?

B10. How can women receive medical care if they need assistance for childbirth at night, on the weekend, or during a holiday?

B11. Are there women who are unable to get to the health service to deliver their babies? In such cases, women ask for help from whom? (For example, traditional birth attendants, traditional healers, midwives, etc)

B12. Is there a travel cost to get to the clinic?

B13. Where do women seek medical care after childbirth?

B14. What are the danger signs for health problems in newborn babies? Where do women seek medical care for their baby?

B15. What are the breastfeeding practices in this community?

B15a. What changes in breastfeeding practices have been evidenced since you arrived in Brazil?

- - - In case there are reports of changes: please can you describe these changes?

B16. What do women in this community do to prevent or postpone pregnancy?

B16a. Where can you find reliable sources of family planning information?

B16b. What family planning methods are available to you at this site?

B16c. What methods would you like to use that is not available?

B16d. What barriers exist to access family planning methods for women who like to use?

B16e. Are there any costs for access to the methods, and if so, what are they?

B16f. What can women do if they have unprotected sex and do not want to get pregnant?

- What have they heard about emergency contraception?

- Do they have access to emergency contraception?

B16g. What do women in this community do if they are pregnant but do not want to become pregnant?

**C. Now we would like to talk about STI/HIV/AIDS for a few minutes.**

C1. In your opinion, what do the people living in the shelter know about HIV/AIDS?

C2. In your opinion what do people do to prevent HIV transmission?

C3. What services are available for people living with HIV in this community?

C4. Are there costs to access these services?

C5. Have you heard of any other diseases that you can get from having sex, for example, sexually transmitted infections? (Probe: Can you name any sexually transmitted infections?)

C6. What would people in your community do if they thought they had a sexually transmitted infection?

C7. In this shelter, are condoms available?

**[If respondents are not sure or say no, skip to question C8c].**

C7a. **IF YES**: Are the condoms free?

C7b **IF YES:** How have women learned about where these condoms can be found?

C7c. **IF YES OR NO**: What are the barriers to easy access to condoms? (Probe: What can be done to make condoms more accessible?)

C.8. Could you describe what you consider to be the best way to provide information about the availability of reproductive health services?

**D. Now, we would like to talk about some issues about violence. Now we would like to ask you some questions about how women who have experienced violence can access care.**

In your opinion, are there women in the shelters who have suffered violence?

Do you know relatives, friends, who experienced situations of violence here in the shelter?

What do you know about violent acts in the shelter?

D1. Since you have migrated, do you know of cases of women who have suffered any kind of violence? [Probe: Physical, psychological, economic, or sexual violence].

D2. In your opinion, to what extent is sexual or other kind of violence perceived as a problem in this community?

D3. What options or services are currently available to women living in this community if they have experienced sexual violence or other kind of violence?

D3a. **If they mentioned knowing women who suffered sexual violence**, ask: Did the women you know who suffered sexual or other kind of violence seek help from these services? What are in your opinion some of the reasons why they have not made use of these services?

D4. Is there any psychological care at the services for people who have experienced sexual or other kind of violence?

D4b. What do you know about the quality of these health services?

D5. In your opinion, what health services should be available to women who experience sexual or other kind of violence?

**E. These final questions refer to the mental health status of migrant women and girls.**

E1. What do you think are the mental health needs of women in this context in which they are living?

E1a. Do you know other migrant women who have felt very nervous, scared, anxious, aggressive, sad or who feel that they cannot continue with their current situation?

E2. How do you think health care should respond to these needs?

**F. Before concluding, I would like to invite you to ask or talk about any reproductive health or women's and girls' health issues that I have not asked you about, but you find interesting to discuss.**

***We thank you for your time. You have all helped us to better understand the situation here. Your contributions are deeply appreciated.***

SPANISH

**PROJETO: “SERVIÇOS DE SAÚDE SEXUAL E REPRODUTIVA PARA VENEZUELANAS NA FRONTEIRA ENTRE VENEZUELA E BRASIL – 2019/2020”.**

**Grupos focales de discusión -Instrumento**

**Mujeres (18-49 años)**

**IntroducCCIÓN**

Antes de iniciar la discusión de los grupos focales, asegúrese de recibir el formato de consentimiento informado.

“Hola, mi nombre es _______________. Yo trabajo para _______________. Este es mi equipo [Introduzca al tomador de notas]. Nosotros estamos llevando a cabo una investigación sobre temas relacionados a la salud y en particular a la Salud Reproductiva en esta crisis humanitaria. El propósito de este grupo focal es conocer más sobre la integración de la salud reproductiva a las respuestas humanitarias que Uds. están recibiendo, así como conocer sus experiencias de acceso a los servicios. Durante la discusión, yo les haré preguntas acerca de sus experiencias con relación a la atención en salud reproductiva. Mis colegas anotarán algunas observaciones y si Uds. están de acuerdo estaremos gravando las respuestas. Sus respuestas serán usadas para un reporte que puede ser publicado o presentado en uno o más foros de salud pública. Su nombre no será incluido en ningún documento o presentación, pero el nombre del lugar en el que nos encontramos sí puede aparecer. No hay ningún beneficio directo por participar en este estudio. Si usted se siente incómoda con cualquiera de los temas abordados en nuestra conversación, puede no participar o dejar de participar en cualquier momento de la discusión. Usted también puede escoger no responder a cualquiera de las preguntas.

***¿Podemos continuar?***

**PREGUNTAS**

1. **En primer lugar, nos gustaría hacerles algunas preguntas generales sobre la situación de las mujeres migrantes en este lugar.**

A1. ¿Qué problemas generan más preocupaciones a las mujeres que están aquí?

A1a. ¿Qué puede hacerse para mejorar estos problemas en particular?

A2. Por favor, pueden contarnos cómo las organizaciones que responden a esta emergencia se han comunicado con la comunidad afectada respecto a la respuesta a la emergencia? ¿Cómo fue el contacto con Uds.? ¿Como se enteraron Uds.?

- ¿En la opinión de Uds., la forma de comunicarse fue adecuada, suficiente, ¿la entendieron?
- ¿Los centros a los que tienen que ir para recibir atención en salud, son de las organizaciones que prestan ayuda/servicios humanitarios o son del municipio?

1. **Ahora, nos gustaría hacerles algunas preguntas generales sobre la prestación de servicios de salud aquí donde Uds. viven ahora**

B1. Desde que llegaron, ¿qué suministros para la menstruación, la higiene o el parto limpio han sido distribuidos a las mujeres y las niñas?

**En caso de que fue distribuido por lo menos alguno de esos suministros:**

B1a. ¿Quién hizo la distribución, ¿dónde fue hecha y cuántas veces se realizó en el último mes?

B1b. ¿Que piensan Uds. sobre esas distribuciones? ¿Qué piensa la comunidad en general sobre esas distribuciones?

- ¿Son suficientes o no?
- ¿Quién decide la cantidad?

**En caso de que no fue distribuido por lo menos algún de esos suministros, indague:**

B1c. ¿Qué razones se les han dado para no tener estos suministros?

**Continuando con la discusión sobre la calidad de los servicios de salud en Boa Vista.**

B2. ¿Cuántas mujeres se han visto involucradas en el diseño o la entrega de servicios para satisfacer las necesidades en salud en este abrigo?

B2a. ¿Con quién se puede discutir si hay necesidades insatisfechas de las niñas/mujeres de este abrigo (Por ejemplo, el líder de la cuadra, el líder del abrigo, la asociación de mujeres, ONG, ONU, etc.)?

B3. ¿Han tenido contacto con servicios de salud en Boa Vista? ¿Cómo ha sido el trato del personal de salud?

B3a. ¿Han sabido o escuchado sobre las experiencias de otras mujeres que han tenido contacto con los servicios de salud de Boa Vista?

B4. ¿Qué programas hay para adolescentes? [**Indague**: ¿alguna vez han escuchado/ visitado un programa específicamente dirigido a los y las jóvenes?]

B4a. ¿Qué servicios de salud reproductiva ofrece este programa?

B5. ¿Cuáles son algunas de las razones por las que una mujer no buscaría servicios de salud?

B5a. (**Indague**: ¿Algún problema con los horarios de la clínica o el hospital, los profesionales o los medicamentos, la edad de la mujer o su estado civil?)

B6. ¿Qué precauciones de seguridad constituyen una barrera para que las mujeres busquen servicios de salud?

B6a. ¿Cuáles son las cosas que preocupan a las mujeres sobre los comentarios que escuchan?

B7. En general, ¿Cómo considera que pueden mejorarse los servicios de salud para las mujeres y jóvenes migrantes?

**Nos gustaría también discutir sobre la atención en salud, especialmente la salud sexual y reproductiva. Empecemos hablando sobre cuestiones relacionadas con el embarazo y el parto.**

B8. ¿Cuándo las mujeres están **embarazadas** en dónde buscan atención médica?

B8a. ¿Cómo tuvieron conocimiento sobre estos servicios para las mujeres embarazadas y para dar a luz? Uds. saben cómo funciona ese acompañamiento prenatal? ¿Todas las embarazadas hacen prenatal?

B8b. ¿Que han oído acerca de la calidad de los servicios para mujeres embarazadas y que van a dar a luz?

B8c. ¿Qué síntomas harían que las mujeres busquen ayuda cuando están embarazadas? (**Indague:** Problemas de salud, signos de peligro). ¿Como consiguen ayuda? (consulta, exámenes)

B9 ¿Dónde buscan las mujeres atención médica **cuando van a dar a luz**? ¿Cómo llegan hasta allí?

B10. ¿Cómo pueden las mujeres recibir cuidado médico si necesitan asistencia para el parto en la noche, durante el fin de semana o durante un día festivo?

B11. ¿Hay mujeres que **no consiguen** llegar al servicio de salud para tener el parto? ¿En esos casos las mujeres piden ayuda a quién? (Por ejemplo, parteras tradicionales, curanderos tradicionales, parteras, etc.)

B12. ¿Hay algún costo de viaje para llegar a la clínica?

B13. ¿Dónde buscan las mujeres atención médica **después del parto**?

B14. ¿Cuáles son las señales de peligro por problemas de salud de los bebés recién nacidos? ¿Dónde buscan las mujeres atención médica **para su bebé**?

B15. ¿Cuáles son las prácticas de lactancia en esta comunidad?

B15a. ¿Qué cambios en las prácticas de lactancia se han evidenciado desde que llegaron a Brasil?

- - - En caso de que refieran cambios: por favor, ¿pueden describir esos cambios?

B16. ¿Qué hacen las mujeres de esta comunidad para prevenir o posponer el embarazo?

B16a. ¿Dónde pueden encontrar fuentes confiables de información sobre planificación familiar?

B16b. ¿Qué métodos de planificación familiar están disponibles para ustedes en este lugar?

B16c. ¿Qué métodos les gustaría usar que no estén disponibles?

B16d. ¿Qué barreras existen para acceder a los métodos de planificación familiar si quieren hacer uso de estos?

B16e. ¿Hay algún costo para el acceso a los métodos? ¿Si los hay, cuales son los costos?

B16f. ¿Qué pueden hacer las mujeres si tienen sexo sin protección y no quieren quedar embarazadas?

- ¿Qué han oído sobre la anticoncepción de emergencia?

- Tienen acceso a la anticoncepción de emergencia?

B16g. ¿Qué hacen las mujeres de esta comunidad si están embarazadas, pero no quieren quedar embarazadas?

1. **Ahora nos gustaría hablar sobre ITS/VIH/SIDA por algunos minutos.**

C1. ¿En su opinión, qué saben las mujeres sobre VIH/SIDA?

C2. ¿En su opinión qué hacen las personas para prevenir la transmisión de VIH?

C3. ¿Qué servicios existen para las personas de esta comunidad que viven con VIH?

C4. ¿Hay costos para acceder a estos servicios?

C5. ¿Ha oído de alguna otra enfermedad que pueda adquirir por tener sexo, por ejemplo, infecciones de transmisión sexual? (**Indague:** ¿Pueden nombrar alguna infección de transmisión sexual?)

C6. ¿Qué harían las personas de su comunidad si piensan que tienen una infección de transmisión sexual?

C7. ¿En qué lugar de este abrigo se pueden encontrar condones?

**[SI las entrevistadas no están seguras o dicen que no, salte a la pregunta C8c]**

C7a. **EN CASO DE QUE SÍ:** ¿Son gratis los condones?

C7b **EN CASO DE QUE SÍ:** ¿Cómo han aprendido las mujeres sobre donde pueden encontrarse estos condones?

C7c. **EN CASO DE QUE SÍ O NO:** ¿Qué barreras dificultan el acceso fácil a los condones? (**Indague:** ¿Qué puede hacerse para que los condones sean más accesibles?)

C.8. ¿Podrían describir cuál consideran que es la mejor manera para entregar información acerca de la disponibilidad de servicios de Salud reproductiva?

1. **Ahora nosotros queremos conversar sobre violência. Ahora queremos hacer algunas preguntas sobre como las mujeres que han sufrido experiencias de violencia pueden tener acceso a los servicios de salud.**

En su opinión, hay mujeres que han sufrido algún tipo de violencia?

Usted conoce parientes, amigos que han experimentado situaciones de violencia aqui en el abrigo?

Qué sabe sobre actos de violencia que han ocurrido aqui en el abrigo?

D1. ¿Desde su proceso de migración conocen casos de mujeres que han sufrido algún tipo de violencia? [Indague: Violencia física, psicológica, económica o sexual]

D2. En su opinión hasta qué punto la violencia sexual u otras formas de violencia son percibidas como un problema en esta comunidad?

D3. ¿Qué opciones o servicios están actualmente disponibles para las mujeres que viven en esta comunidad en caso de que hayan sufrido violencia sexual u otro tipo de violencia?

D3a. **Si mencionaron conocer mujeres que sufrieron violencia sexual, indague**: ¿las mujeres que Uds. conocen y que sufrieron violencia sexual u otro tipo de violencia buscaron ayuda en estos servicios? ¿En su opinión cuáles son algunas de las razones por las cuales ustedes creen que ellas no han hecho uso de estos servicios?

D4. ¿Quién presta servicios de atención psicológica en esos servicios para personas que han experimentado violencia sexual u otro tipo de violencia?

D4b. ¿Qué saben sobre la calidad de estos servicios de salud?

D5. In your opinion, what health services should be available to women who experience sexual or other kind of violence?

D5. ¿En la opinión de Uds. qué **servicios de** **salud** deberían estar disponibles para las mujeres que experimentan violencia sexual u otro tipo de violencia?

1. **Estas preguntas finales hacen referencia al estado de la salud mental de las mujeres y las niñas migrantes.**

E1. ¿Cuáles creen que son las necesidades de salud mental de las mujeres en este contexto en el que están viviendo?

E1a. ¿Conocen a otras mujeres migrantes que se hayan sentido muy nerviosas, asustadas, ansiosas, agresivas, tristes o que sientan que no pueden continuar con su situación actual?

E2. ¿Cómo consideran que debería responder la atención en salud a esas necesidades?

1. **Antes de finalizar, me gustaría invitarlas a preguntar o hablar sobre cualquier tema de salud reproductiva o salud de las mujeres y las niñas, por el cual yo no les haya preguntado, pero a ustedes les parece interesante discutir.**

*Les agradecemos por su tiempo. Todas ustedes nos han ayudado a entender mejor la situación aquí. Sus contribuciones son profundamente apreciadas.*
